# 

**Published:** 1869-11

**Authors:** 


THREE DOLLARS A YEAR. SINGLE COPIES, 30 CTS.

VOLUME XXVI. *NOVEMBER*, 1869. NUMBER XX

THE

### Chicago Medical Journal,

A MONTHLY RECORD OF

*Medicine, Surgery & Collateral Sciences.*

J. ADAMS ALLEN, M.D., LL.D., WALTER HAY, M.D.,
EDITORS.

CHICAGO:

W. B. KEEN & COOKE, PUBLISHERS,

113 & 115 STATE STREET.

To whom communications for the Editors and books for review must be addressed.
*The postage on this Journal is 24 cents a year—payable quarterly in advance.*

Church, Goodman & Donnelley, Printers.

